# Human evolution in Siberia: from frozen bodies to ancient DNA

**DOI:** 10.1186/1471-2148-10-25

**Published:** 2010-01-25

**Authors:** Eric Crubézy, Sylvain Amory, Christine Keyser, Caroline Bouakaze, Martin Bodner, Morgane Gibert, Alexander Röck, Walther Parson, Anatoly Alexeev, Bertrand Ludes

**Affiliations:** 1 Laboratoire AMIS, FRE 2960 CNRS, Université de Toulouse, 37 allées Jules Guesde, 31073 Toulouse, France; 2 Laboratoire d'Anthropologie Moléculaire, Institut de Médecine Légale, Université de Strasbourg, 11 rue Humann, 67085 Strasbourg, France; 3 Institute of Legal Medicine, Innsbruck Medical University, 6020 Innsbruck, Austria; 4 Institute of Mathematics, University of Innsbruck, Technikerstrasse 13, 6020 Innsbruck, Austria; 5 Presidency of the University, Yakutsk University, Sakha Republic, Russia

## Abstract

**Background:**

The Yakuts contrast strikingly with other populations from Siberia due to their cattle- and horse-breeding economy as well as their Turkic language. On the basis of ethnological and linguistic criteria as well as population genetic studies, it has been assumed that they originated from South Siberian populations. However, many questions regarding the origins of this intriguing population still need to be clarified (e.g. the precise origin of paternal lineages and the admixture rate with indigenous populations). This study attempts to better understand the origins of the Yakuts by performing genetic analyses on 58 mummified frozen bodies dated from the 15^th ^to the 19^th ^century, excavated from Yakutia (Eastern Siberia).

**Results:**

High quality data were obtained for the autosomal STRs, Y-chromosomal STRs and SNPs and mtDNA due to exceptional sample preservation. A comparison with the same markers on seven museum specimens excavated 3 to 15 years ago showed significant differences in DNA quantity and quality. Direct access to ancient genetic data from these molecular markers combined with the archaeological evidence, demographical studies and comparisons with 166 contemporary individuals from the same location as the frozen bodies helped us to clarify the microevolution of this intriguing population.

**Conclusion:**

We were able to trace the origins of the male lineages to a small group of horse-riders from the Cis-Baïkal area. Furthermore, mtDNA data showed that intermarriages between the first settlers with Evenks women led to the establishment of genetic characteristics during the 15^th ^century that are still observed today.

## Background

The Yakuts (or Sakhas) from the Republic of Sakha are of particular interest because they contrast with other populations from Siberia in many respects such as in their specific funeral practices. Before Christianization, part of the population was buried, which is exceptional in this region of the world as most bodies were put in aerial graves on platforms or on trees. At the same time, the Yakuts are considered as the most remarkable example of northward expansion into Siberia [1] and especially as these semi-nomadic herders contrast so strikingly with surrounding Siberian populations on the basis of their Turkic language and their horse- and cattle-breeding economy. The geographic specificities of Central Yakutia, favorable to the development of a pastoralist economy and reflecting the environmental conditions of the South, certainly initiated the development of the Yakut culture in this region. Although several hypotheses regarding the geographic origin of this atypical population have been proposed from results of the first archaeological investigations of AP Okladnikov [2] to the latest population genetic studies [3-6], the precise origins of the Yakuts and their admixture with the indigenous tribes of Siberia, especially the Tungus, have been debated. Nevertheless, archaeological data currently agree with the appearance of the Yakut culture around the 14^th ^century A.D. [7].

Recent population genetics data tend to support a dual origin for the maternal lineages of the Yakuts and admixture with the Tungus, and demonstrate that the paternal lines observed today result from a strong bottleneck that led to the restriction of these lineages in the Yakut population [3,4,6]. This does not, however, address the question of the number of settlers and the precise origin of the paternal lineages, or the admixture rate with indigenous populations [6]. Furthermore, the demographic changes occurring in the Yakut population since the Russian colonization make it difficult to reconstruct the genetic history using present day samples. Russians came into contact with the Yakuts in 1632. Christianization began around 1760 and by the beginning of the 19^th ^century, a Christian ritual was performed in the majority of burials. From the 17^th ^to the 19^th ^century smallpox and measles epidemics, caused by contact with Russians settlers, decimated the Yakut people and their neighbors, and during the Second World War a large proportion of young Yakut males was killed. Therefore ancient DNA (aDNA), which allows the direct study of an ancient population without their descendants, represents a major advantage in order to avoid the possible bias represented by recent genetic events [8,9].

**Figure 1:**
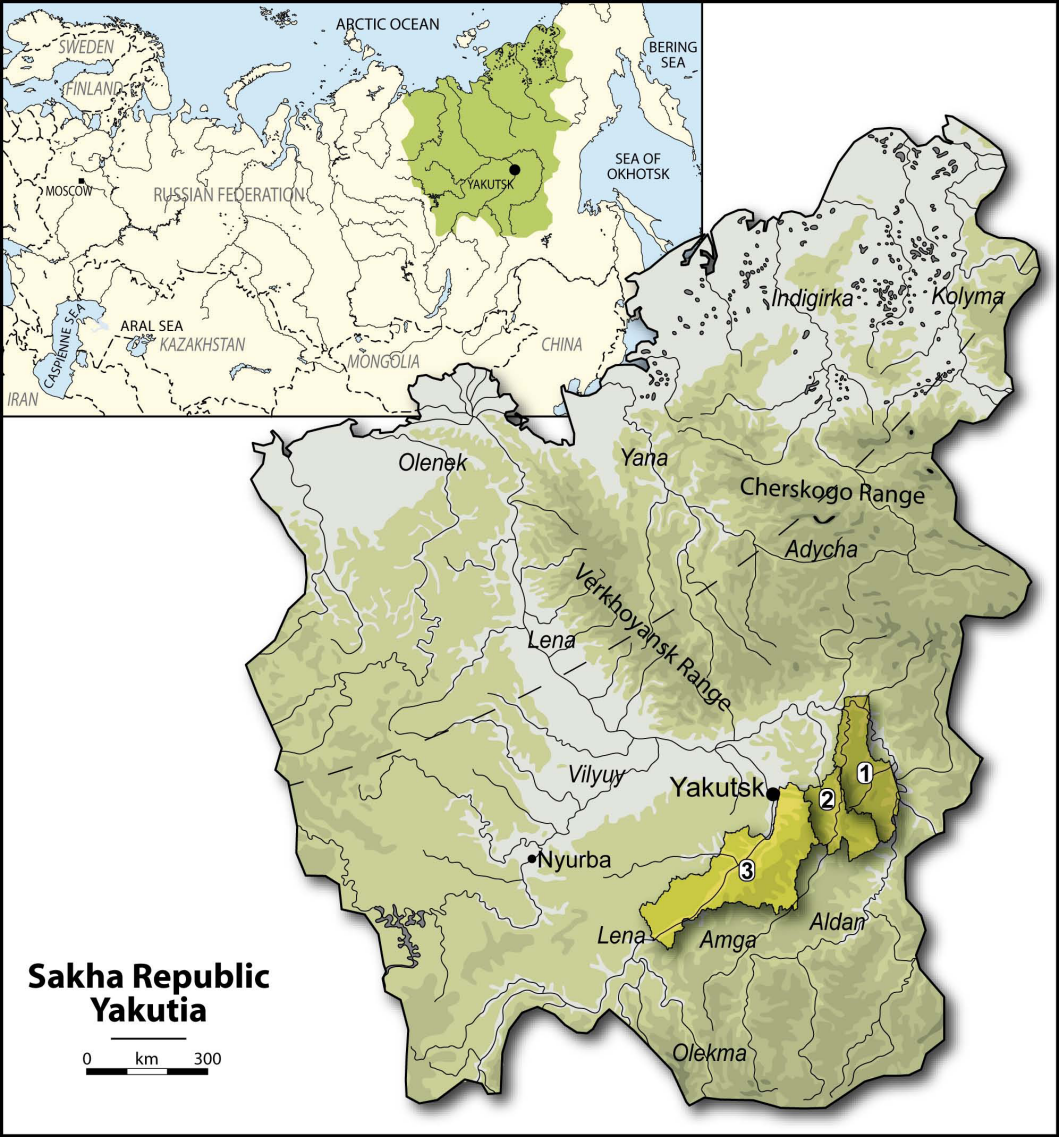
**Location of Yakutia and its districts**. Geographic location of the three districts in Central Yakutia where the excavations were performed. 1: Tattinsky, 2: Churapchinsky, 3: Khangalassky.

**Figure 2:**
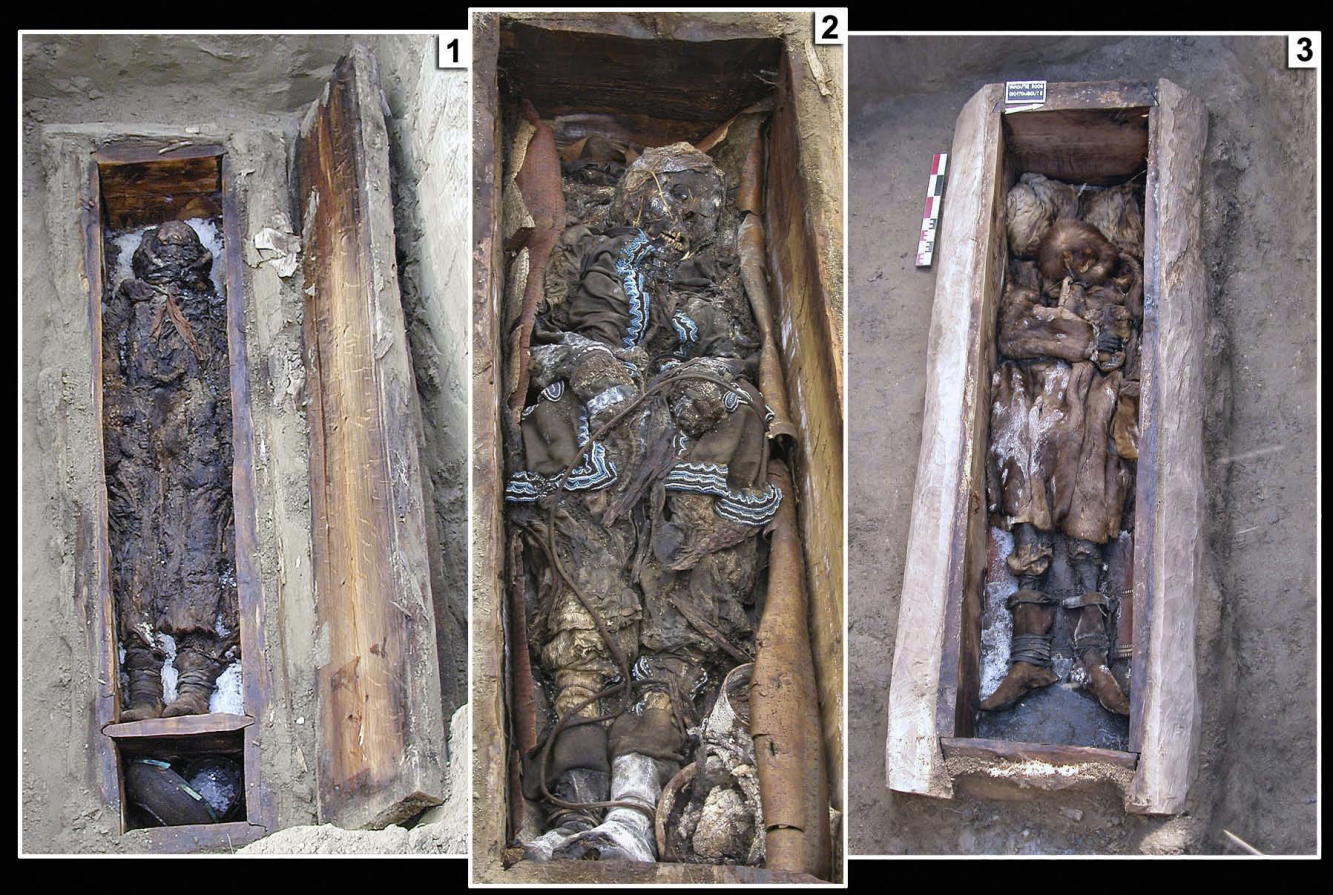
**Frozen bodies**. Three frozen bodies from Yakutia a few minutes after the opening of the graves [57]. 1: Kémus Buluussé (Khangalasssky), 2: Kyys Ounouoga (Churapchinsky), 3: Okhtoubout 3 (Churapchinsky).

This study adopts a population genetics approach to an ancient sample composed of 65 individuals from Central Yakutia in order to address the questions detailed above. Human aDNA studies' outcome quality is closely related to the preservation of DNA and to the absence of potential contaminations (Figure 1). The environmental conditions present in Yakutia, one of the coldest parts of the world, are generally favorable to a good preservation of nucleic acids. Indeed, the exceptional preservation state of most of the excavated graves enabled us to successfully analyze several samples collected from 58 frozen bodies or skeletons from the last six centuries (Figure 2).

## Results

### Nuclear DNA quantification

The results of the nuclear DNA quantification are summarized in Table 1. The purification protocol was efficient in removing inhibitors in all except six bone extracts. In order to avoid the effect of PCR inhibitors, the samples YAKa33, 36, 69, 70 and 79 were diluted by a factor of 1/2 to 1/5 and successful amplifications were subsequently obtained. For the sample YAKa28 it was not possible to remove the inhibitors and therefore no results were obtained for further amplifications. No inhibitors were detected in the teeth or hair extracts. Moreover, the DNA extracts obtained from teeth showed the highest mean concentration of nuclear DNA and the highest frequency of samples yielding a concentration superior to 100 pg.μl^-1^.

**Table 1:** Summary of nuclear DNA quantification by real time PCR depending on different substrates.

	Substrates		
	
	Bones	Teeth	Hairs
Number of samples	64	18	12

Number of DNA extracts quantified	133	18	41

Mean quantity of DNA (minimum-maximum observed)	0.23 ng.μl^-1^ (0-2.32 ng)	0.56 ng.μl^-1^ (0.003-1.54 ng)	0.12 ng.μl^-1^ 0-0.945 ng)

Frequency of DNA extracts yielding a DNA concentration > 500 pg.μl^-1^	18.86%	50%	7.30%

Frequency of DNA extracts yielding a DNA concentration comprised between 500 and 100 pg.μl^-1^	18.86%	38.90%	19.50%

Frequency of DNA extracts yielding a DNA concentration below the detection limit	20.30%	5.5%	19.50%

Frequency of DNA extracts containing PCR inhibitors	8.27%	0	0

### Y-chromosomal analyses

The multiplex amplification of the Y-chromosomal STRs was successful for 27 of the 38 (71%) male samples, where full profiles or profiles with only one missing marker were obtained (See Additional file 1). The lowest amplification success rate (69%) was observed for the DYS392 locus that is the largest marker included in the STR panel. All loci with a length over 200 bp showed more than 20% amplification failures. This molecular behavior is a well known phenomenon and has been described as an effective way to ensure the authenticity of results [10].

Twelve different haplotypes were observed. Nine were unique, one was common to two individuals (YAKa17 and 19) and two haplotypes were widely shared among our ancient sample set. These two haplotypes were present in eight (Ht1: YAKa15, 21, 29, 31, 34, 39, 40, 41) and seven individuals (Ht2: YAKa64, 66, 67, 68, 69, 70, 78), and differed from each other by only one mutation step at the loci DYS389II and DYS392. Therefore, Ht1 and Ht2 could share a recent common ancestor, since two mutation events in a single father/son transmission have been previously described [11]. Besides, Ht1 and Ht2 were the most frequent lineages in our present day sample set (Ht1: 16.5% and Ht2: 27.8%) as well as in the data published by Pakendorf et al [6] (Ht1: 43% and Ht2: 10%).

The genetic diversity indices (Table 2) show equivalent values for the ancient and the present day sample sets of our study. Surprisingly, the diversity observed in our Central Yakut sample set is higher than the one presented by Pakendorf et al [6] although the samples were collected in the same area.

**Table 2:** Y-STR diversity indices for the three Yakut sample sets

	**N**^**2**^	**n**^**3**^	Haplotype diversity	**MPD**^**4**^	**SE**^**5**^	Gene diversity	SE
Ancient Yakuts	25	12	0.89	3.39	1.79	0.308	0.182

PD Yakuts ^1^	97	44	0.90	3.49	1.79	0.218	0.124

Central Yakuts	183	44	0.79	2.07	1.16	0.23	0.14

^1 ^Present day Yakuts, ^2^Number of samples, ^3^Number of haplotypes, ^4^MPD: mean pairwise differences, ^5^Standard error. Central Yakuts [8]

Sixty one percent (8 out of 13) of the haplotypes (Ht1, Ht2, Yaka56, 65, 71, 80, 81, 86) were affiliated to the N1c (TAT-C) haplogroup on the basis of the SNP analyses. This haplogroup is considered as the most frequent in the Yakut population, and its frequency varies across studies from 75% [12] to 100% [13]. Sample YAKa26 was affiliated to haplogroups K. The SNP typing was inconclusive for 5 individuals (YAKa17, 19, 47, 49 and 57); nevertheless the affiliation to N1c was excluded on the basis of the absence of the TAT-C mutation.

Although all the Y-STR haplotypes have been transmitted to the present day Yakuts, only a few of them are shared with other populations. A comparison to the YHRD database and other literature data showed that only Ht1 and the haplotype of the YAKa26 individual matched with Evenk male lineages; confirming the specificity of the N1c lineages. In this case, one can assume that N1c comes from Yakut ancestors considering the low N1c frequency in Evenks [6,14]. Moreover, the ethnical affiliation of one individual to either the Yakut or the Evenk population could be difficult nowadays, considering that these two ethnic groups share a similar lifestyle or language in some areas of Yakutia. The Y-STR haplotypes of YAKa17 and YAKa26 were found in present day Mongols, Buryats, Kalmyks and Central Asians.

**Figure 3:**
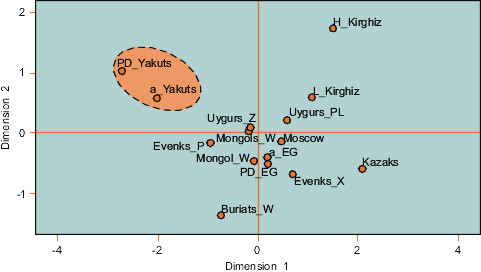
**MDS plot of genetic distances between populations on the basis of Y- STRs**. MDS plot based on pairwise Fst values calculated from Y-chromosomal STRs. a_Yakuts: ancient Yakuts, PD_Yakuts: present day Yakuts (97 samples, data not shown, available upon request), Buryats_W: Buryats [75], Evenks_P: Evenks [50], Evenks_X: Evenks [76], a_EG: ancient Mongols from Egyin Gol [47], PD_EG: present day Mongols from Egyin Gol [48], Kazakhs [77], H_Kirghiz and L_Kirghiz: Kirghiz [77], Mongols_K: Mongols [78], Mongols_W: Mongols [75], Moscow: Russians (Jobling, YHRD database: http://www.ystr.org/index.html), Uyghurs_PL: Uyghurs [77], Uyhgurs_Z: Uyghurs [79].

The MDS plot based on pairwise Fst values clearly shows the separation of the Yakuts from all the other populations, since the ancient and present day Yakuts are clearly isolated (Figure 3). Indeed, the Fst distance computations revealed significant differences for each comparison excepted for the two Yakut sample sets.

**Figure 4:**
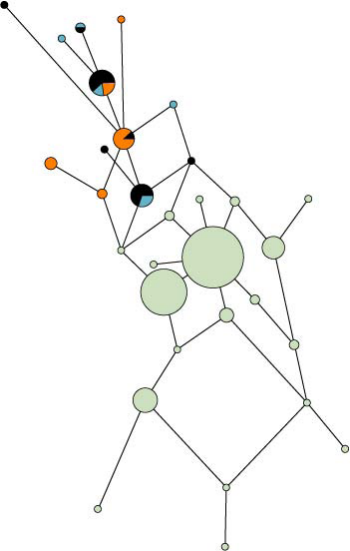
**Median Joining network based on Y-chromosomal haplotype**. Median Joining network based on Y-chromosomal haplotypes belonging to the haplogroup N1c. Black: ancient Yakuts, Blue: present day Yakuts, Orange: Evenks. Green for Other populations included in the computations: Buryats [75,80], Evenks [80,81], Mongols [76,80], Tuvans [43,80], Uyghurs [76,80] and Altaians, Evens, Kalmyks, Khakassians, Koryaks, Shors, Sojots, Tofalars, and Yakuts from Derenko et al. [80].

The median network based on the Y-STR haplotypes belonging to the N1c haplogroup is undoubtedly affected by sample sizes. Nevertheless, the separation between the Yakuts and the other populations is visible (Figure 4). Indeed, both ancient and present day Yakuts are grouped in one isolated cluster together with the Evenks. The different haplotypes of this cluster are separated by one mutation step (except YAKa56), indicating that these closely related paternal lineages have emerged from a reduced number of individuals in a short period of time. This observation is consistent with earlier studies [6,15].

### Mitochondrial DNA analysis

The HVS-1 sequences were unambiguously validated for 60 individuals (See Additional file 2). Five subjects were excluded from the analysis: YAKa28, 42, 48, 49 and 72. No amplification was obtained for the sample YAKa28 due to the presence of inhibitors in the DNA extracts. Amplification failures were also observed for the sample YAKa42 and only one of the two sub-regions of HVS-1 was successfully amplified for sample YAKa72. Finally, multiple heteroplasmies were observed in the electropherograms of YAKa48 and 49. The fact that these two samples were collected in museums, increasing the risk of pre-laboratory contamination, led us to exclude these two subjects from our study.

Two heteroplasmic positions were observed for three distinct samples. One T/C heteroplasmy at position 16093 was observed in the samples YAKa20 and YAKa39 in teeth and hair DNA extracts only. This heteroplasmic position is observed in the present day Yakut population (this study; [6]) and is also well known as a hotspot of post-mortem damage [16]. The second heteroplasmy was observed at position 16293 in the sample YAKa58. This position has been reported as a potential post-mortem mutation [16]. Therefore, these heteroplasmic sites were treated as missing data for these specific samples in all the subsequent analyses.

Gene diversity in our ancient sample was calculated using the ARLEQUIN 2.001 program and then compared with different populations. Both ancient and present day populations from Central Yakutia showed a gene diversity of 0.9627 (+/- 0.0117). This value is in agreement with the data from [6]. We observed 30 different haplotypes among the 60 studied individuals, and 19 were found only once. Twenty nine haplotypes were assigned to different haplogroups with the near matching method. The subject YAKa83, plausibly a young shaman from the early 18^th ^century, was affiliated to haplogroup H6 due to the typing of coding region SNPs [17] since the assignment on the basis of the HVS-1 haplotype was inconclusive.

The overall haplogroup distribution (Table 3) in our ancient sample set corresponds with earlier studies focused on the present day Yakuts [6,18-20]. As can be seen in Table 3, the ancient Yakuts present high frequencies of haplogroups C and D. Haplogroup C and specifically the lineage C4 represents the most abundant haplogroup in our ancient sample set with a frequency of 30%. The highest frequency of this lineage is observed in the Evenks, but C4 is also prevalent in South-Western Siberian populations such as Tuvans, Buryats and Tofalars [15,21]. Among haplogroup D, the D5 lineage is present in seven out of 13 individuals and represents 12.5% of the ancient sample set (the analysis of the autosomal STRs revealed that YAKa34, 35, 36, 37 and 38 shared close familial relationships, therefore only one individual was included in the calculations). This sub-haplogroup is usually present at low frequency in Siberian populations [15,21,22] and in Central Asian or Mongolian populations [23,24]. Interestingly, the two most frequent D5 haplotypes seem to share a recent common ancestor as they differ only by one transition at position 16172. This high frequency of subhaplogroup D5 has been regarded as specific to the Yakut maternal lineages by Pakendorf et al. [6]. The Buryat ascendance of the D5 lineages demonstrated in their analyses was not clearly supported by our ancient DNA data.

**Table 3:** MtDNA haplogroup frequency distribution in our ancient and present day Yakut sample sets and in the Yakuts from Central Yakutia [8]

Haplogroup	Ancient Yakuts	PD Yakuts	Central Yakuts
A	3.57	2.41	2.2

B	5.36	1.81	4.4

C	42.86	46.99	38.5

D	30.2	29.52	33

D/G	1.8	1.2	

F	3.57	3.61	6.5

G			3.3

H	1.8	1.20	1.1

J	3.57	2.41	1.1

K	1.8	0.6	1.1

M	1.8	3.01	1.1

N			

T		1.81	1.1

U		1.81	2.2

V			

W	1.8		1.1

X			

Y	1.8	0.6	2.2

Z		0.6	1.1

Sub-haplogroup frequencies have been merged under the name of the basal haplogroup to simplify the comparison. Frequencies are given in percent.

The sequence of YAKa23 belonged to haplogroup A4b, that is present at low frequencies in Western and Middle Siberian populations such as the Mansis, Tuvans and Evenks [21,22]. YAKa78 was assigned to the novel haplogroup A8, found in the Kalmyks and Buryats [15]. Two haplotypes were assigned to haplogroup B: YAKa24 to subhaplogroup B4, which is dispersed throughout Mongolia and China [25,26] and the sequences of YAKa52 and YAKa53 to subhaplogroup B5 due to the presence of transitions at positions 16111, 16140, 16189 and 16243, also present in one Buryat studied by Derenko et al [27].

Two sequences belonged to the F1b lineage (YAKa22 and YAKa68), widespread in populations from South Siberia [27]. The haplotype of YAKa71 was affiliated to the M13a subhaplogroup present in Buryats, Khamnigans [15] and Mongols [28].

The proportion of the sequences affiliated to the West Eurasian haplogroups (H, J, K, T and W) was low in our ancient sample set, as the merged frequencies corresponded to 8.3% (5 samples). The sequence of YAKa83 was affiliated to subhaplogroup H6 which is characteristic for Central Asians [29] and less frequent in Western Europe. These sequences were observed in samples distributed in the second and third period, and no increase in the frequency of the West Eurasian lineages could be noted for the period following the Russian colonization. Besides, these haplogroups are observed at similar frequencies in various Siberian populations [21,27].

The size of the sample set should be considered as a potential source of bias in the overall haplogroup distribution. Nevertheless, we observed the same distribution as was found in the present day population. This statement has been verified by the calculation of the Fst distances since there are no significant distances between the Yakuts from the pre-colonization era and the ones from the Christian and present day periods.

In an attempt to trace the origins of the ancient mtDNA lineages, each haplotype was compared to data from the literature (See Additional file 3). This comparison showed that more that 73% of the haplotypes found in our ancient samples have been transmitted to the present day Yakut population. Among the nine haplotypes absent in the present day Yakuts, four were not found in any of the populations included in the database due to their unique combination of polymorphisms or private mutations. The haplotypes belonging to haplogroup C are shared with various populations but most specifically with Buryats (6 out of 11 haplotypes), Evenks (5 out of 11) and Tuvans (6 out of 11). On the contrary the sequences assigned to haplogroup D appear to be more restricted to the Yakut population. This specificity is even higher for the D5 lineages since they are shared only with two Yakut speaking Evenks, one Buryat, one Mongol and one Daur.

To evaluate if this comparison could bring some information on the different migration waves which led to the formation of the Yakuts, we took the chronological repartition of the samples into consideration. Unfortunately, this approach failed to reveal any clear pattern, since individuals from different periods were distributed among all the haplogroups and matched to populations without any identifiable preference.

**Figure 5:**
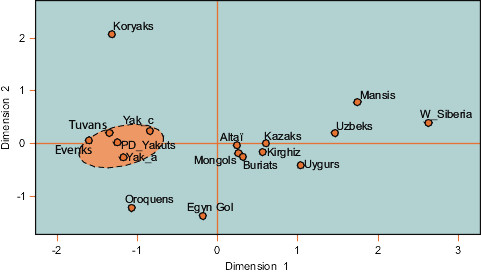
**MDS plot of genetic distances between populations on the basis of mtDNA**. MDS plot based on Fst values of mtDNA HVS-1 sequences. Yak_a: ancient Yakuts, Yak_c: ancient Yakuts from the Christian period, PD_Yakuts: present day Yakuts (this study). Altaï [82], Buryats [4], Evenks [50], Egyin Gol: [48], Kazakhs [23], Kirghiz [23], Koryaks [83], Mansis [22], Mongols [48], Oroqens [65], Tuvans [3], Uyghurs [23,26], Uzbeks [84], W_Siberia: West Siberians [85].

The MDS plot based on Fst distances (Figure 5) revealed, as for the Y-chromosomal STRs, a high stability through time of the maternal lineages. Indeed, the distances between the chronological groups were not significant. Besides, the Fst values confirm the previous statements based on the comparison of individuals since non-significant distances were observed between ancient Yakuts, Evenks and Tuvans.

In order to clarify the previously obtained results, migration rates were computed with the MIGRATE software. The populations used for the analyses were selected taking the previous results into consideration. To avoid a potential bias related to the sample size, ancient Yakuts from different periods were merged into one group. As mentioned earlier, this approximation should not be deleterious since the two groups showed non-significant genetic distances. The results are presented in Table 4 and indicate that the major genetic contribution to the maternal lineages of the Yakuts came from the Evenks. This observation could be explained by the fact that new settlers were more susceptible to admixture than the autochthonous population due to their reduced number and to their cultural practices (e.g. paternal exogamy). This assumption is also corroborated by the higher genetic diversity of the ancient Yakuts compared to the Evenks and by the important number of mtDNA lineages shared with this population.

**Table 4:** Migration rate estimates between ancient Yakuts, Buryats, Mongols Evenks and Tuvans.

		Values of 2 mN for each recipient group				
**Source group**	**Theta**	**Yakuts**	**Buryats**	**Mongols**	**Evenks**	**Tuvans**

Yakuts	0.00840	_	243.3004	304.1255	395.3631	425.7757
Buryats	0.02018	161.7353	_	661.6444	235.2513	514.6123
Mongols	0.01646	134.2355	958.8253	_	326.0006	479.4127
Evenks	0.00168	665.2773	498.9580	498.9580	_	1330.5546
Tuvans	0.01299	355.7545	889.3863	919.0325	1067.2636	_

### First presence of the Yakutian ancestors and demographic data

The first Yakut graves discovered are dated from the 15^th ^and 16^th ^century. From this period until 1689, only male graves with horse riding equipment (children and adults) and weapons from the steppe area (in most adult ancient graves) are found. The funerary ritual was influenced more by Turkish than by Mongolian culture (although this changed at the beginning of the 18^th ^century). It is unlikely that these graves are the first graves of the new settlers, and it is more probable that this period corresponds to the first time with a relatively high population density. During this early period, Y-chromosomal Ht1 and Ht2 are already present. Simulations (Table 5) suggest two possibilities: either a very small group arrived around the 10^th ^century, or a more important one arrived between the disintegration of the Mongol empire (around 1294 AD) and the discovery of the first graves.

**Table 5:** Simulation of growth per year under a logistic growth model

Percentage of growth/year	0,60%	0,90%	1,50%	1,75%	2%	3%
AD 800	55 people	58 people				
AD 1000	100	140	depleted	depleted	depleted	
AD 1294	5500	4000	300	120	50	depleted
AD 1400	10000	5000	1400	700	400	50
AD 1632	40000	40000	40000	40000	40000	40000

40,000 people is an historical estimation for 1632 AD and we have made estimation for different steps: the beginning of the 9^th ^century, which is a period of an important Mongolo-Turkish expansion with some contacts between these populations and Yakutia [1], 1294 AD which corresponds to the disintegration of the Mongol Empire with the dispersion of many nomad groups, and 1400 AD, a date prior to the most ancient Yakut graves excavated.

### Authenticity of sequences and profiles

The possibility that our data arose from contaminated DNA was considered highly unlikely, since morphological and molecular typing results for sex determination were always in accordance with each other. Moreover, no contamination was observed in the extraction or the PCR blanks. Reproducible PCR results were obtained from multiple extractions and amplifications of the same samples performed at different times.

The choice of autosomal STR markers as a first approach for analyzing the ancient Yakut remains was based on their ability to detect degraded and/or contaminated DNA. 57 out of the 65 human samples yielded amplifiable DNA for more than two markers, but only 20 of the genetic profiles were complete (See Additional file 4). The loci D18S51, CSFP1O, D7S820 and D2S1338 were often not amplified, probably because they are expressed in the higher molecular weight range. Such an inverse dependence of the amplification efficiency on the size of the DNA fragment to be amplified is typical of DNA retrieved from ancient remains and results from the degradation of the DNA [10].

Various factors are thought to affect the success of DNA analysis from ancient tissues: (i) the time from excavation [9,30,31], as post-excavation storage conditions are less favorable for DNA preservation than pre-excavation condition, (ii) the cortical thickness of long bones which favors good DNA preservation (cf. the case of horses) and (iii) the age-at-death of the individual, as it is more difficult to obtain profiles from newborn compared to adult bone. Thus, as time passes after excavation, one would expect a reduction in the number of endogenous DNA and an increase in the number of exogenous contaminant template DNA molecules extracted from a tissue sample, with additional difficulties for newborn and young children compared to adult samples. Of the ten human samples that yielded no STR profiles, four came from the museum and six from frozen burials including four newborn samples.

## Discussion

During the last few years, several studies have focused on the Yakuts using genetic analyses to trace their origins [4-6,20,32-36]. However, to date the use of ancient DNA has been limited due to small numbers of individuals sampled [37,38], in spite of the exceptional taphonomic conditions of Siberia and the possibility of retrieving high quality DNA from remains buried in the permafrost [39,40]. Therefore, this study represents the first attempt to trace the origins of the Yakut population by ancient DNA analyses.

Out of the 65 ancient individuals analyzed in this study, 27 complete Y-chromosomal STR haplotypes were obtained from 38 male individuals, and mtDNA sequences were validated for 60 individuals. The quality of the results obtained in our study may be explained by the presence of permafrost which favors the preservation of DNA [41]. The excavation strategy and the absence of any post-excavation treatment prior to laboratory analyses might have played an important role in the quality of DNA and confirms the statements of Pruvost et al. [31]. Besides, this limited handling of the ancient remains represents a key factor in the reduction of contamination risk by modern DNA. The combination of the precautions against contamination and the validation strategy (i.e. comparison between the profiles of the samples and the researchers, analyses of different substrates, multiple analysis of one sample, phylogenetic validation of the mtDNA sequences) validate the data presented here, even if not all the traditional criteria [42] could be met due to the large number of samples.

The Y-chromosome results obtained from our ancient sample are consistent with earlier studies [6,43]. The specificity of the male lineages was confirmed by statistical analyses (MDS plot, MJ network) and by individual comparison of the Y-STR haplotypes with the literature data since only three haplotypes were shared with other populations. The high frequency of the haplogroup N1c, which is present from the most ancient graves, and the non-significant Fst values between ancient and present day Yakuts, indicates their stability through time. Thus, this genetic result corroborates the assumption that the Yakuts are a highly homogenous population not only geographically [6] but also from a chronological standpoint.

The origin of the most frequent Y-chromosomal haplotypes (Ht1 and Ht2) was difficult to establish on the basis of genetic information. Indeed, these two lineages belonging to haplogroup N1c seem to be restricted to Yakut populations, and were probably present since the period they were first located in Central Yakutia. Interestingly, the comparison with archaeological data revealed that the male individuals (YAKa34, 39, 40, 69, 78) at the beginning of the 18^th ^century, identified as Clan Chiefs (or *tojons*) on the basis of their grave goods (weapons, jewelry, silk clothes, richly ornamented saddles and signet rings), belonged to these two haplotypes. Therefore, archaeological data could bring interesting information in tracing back the origin of these enigmatic male lineages. Indeed, the grave goods of the 15^th^/17^th ^centuries (weapons and horse harnesses) and the construction of coffins with an empty trunk from the 18^th ^century are similar to the burial customs of the Cis-Baïkal area [44] and of the Egyin Gol Necropolis during the 3^rd ^century BC [45-47]. This suggests that the male ancestors of the Yakuts were probably formed of a small group of horse-riders originating from Northern Mongolia or the Baïkal Lake. Later, the patrilineal clan organization of the Yakuts would have facilitated the diffusion of male lineages borne by the individuals of high social rank. This hypothesis is supported by the fact that the Y-chromosomal haplotypes identified by the warriors of the 15^th^/17^th ^centuries were transmitted to the *tojons *of the 18^th ^century. After Russian colonization, the influence of the *tojons *was strengthened by the decrees made by the Empress Anna Ivanovna [1], and this official reinforcement of their power might have increased the spread of a limited number of Y-chromosomal lineages for a second time. Therefore, the early founder effect combined with the Yakutian traditions and the Russian influence would have led to the present day genetic pattern observed for the paternal lineages.

Conversely, the results obtained from the mtDNA analyses revealed a more important diversity and varying origins of the maternal lineages. Indeed, the gene diversity observed in our ancient sample is intermediate compared with the diversity found in small and isolated Siberian groups such as the Chukchi and Mongolian [15,48], Buryatian [4] or Central Asian [23] populations. The haplogroup distribution observed in our sample matched that found in present day Yakuts and is constituted by distant haplogroups found in Siberian, Mongolian and Central Asian populations. The major haplogroup, C4, indicates more specific affinities with the Evenks [21]. Nevertheless, the individual comparison of maternal lineages with data from literature allowed us to precise that four out of the six lineages belonging to the C4 haplogroup were present in the Buryat population. Therefore, the Buryat origin of some of the D5 sub-haplogroups found in the Yakuts as demonstrated by Pakendorf et al. [6] could be expanded to the C4 haplogroup.

The influence of both Buryat and Evenk populations is clearly visible in the mtDNA lineages of the ancient Yakuts. Concerning the contribution of the Evenks to the Yakut's mtDNA gene pool, the admixture might have mostly occurred between Yakut men and Evenk women (as assumed by Pakendorf et al. [6]) according to the high frequency of the C4 haplogroup in these two groups, and since the Y-chromosomal lineages are highly specific to the Yakuts and the genetic diversity indices are lower for Y chromosome than for mtDNA. Moreover, the number of first settlers who arrived in Central Yakutia was certainly limited and the patrilocal exogamy practiced by the traditional Yakut society [49] corroborates the inclusion of autochthonous women. This assumption is further confirmed by linguistic data that reveals intermarriages between the Yakuts and Evenks [50].

Finally, the stability through time demonstrated by the Y chromosome is also observed in the maternal lineages. Indeed, most of the sequences present in our ancient sample (83%) have been transmitted to the present day population. However, this could signal a small loss in variation over the two last centuries, which could be associated with stochastic processes linked to demographic changes undergone by the Yakut population (plague, smallpox) or other phenomena [51-53]. Besides, the Yakuts from all chronological periods are grouped together in the MDS plot and the Fst calculation revealed the absence of significant distances between them. Thus, even if the Russian colonization had an important impact on the expansion of the Yakuts throughout Yakutia [1], its genetic influence appears to be relatively low.

Based on the analyses of the maternal and paternal lineages of ancient Yakuts, we were able to demonstrate that the formation of this population started before the 15^th ^century, with a small group of settlers composed of horse-riders from the Cis-Baïkal region and a small number of women from different South Siberian origins. These assumptions are in agreement with archaeological data [54,55], the founder effect dates calculated by Pakendorf et al. [6] and with the recent coalescent simulations of Yakut mtDNA variation by Zlojutro et al. [56]. The early expansion of the Yakuts was accompanied by intermarriages between Yakut men and Evenk women as evidenced by the migration rates. Our data also suggest that the genetic characteristics of the Yakuts were already well established in the Central Yakutian population during the 15^th ^century and have remained stable until the present day.

## Conclusion

There are few regions in the world where the microevolution of a population over several centuries, based on Y-chromosomal and mitochondrial DNA analyses, can be understood. It is the case, however, in Siberia, notably in Yakutia, where frozen bodies facilitate the exceptional preservation of biological samples. In this article we compared 65 individuals from Central Yakutia: 58 frozen bodies from the 15^th ^to the late 18^th ^century which were excavated over three years, and seven bodies of a later period from more ancient excavations, with 166 individuals from a well-defined contemporary population.

Data authenticity from the frozen bodies was determined by the concordance of morphological and molecular typing for sex determination, reproducible PCR results from multiple extractions, and the amplification and analysis of autosomal STR markers based on their ability to detect degraded and/or contaminated DNA. If the time from excavation and post-excavation storage conditions were less favourable for DNA preservation, age at death also plays a role, as it is more difficult to obtain profiles from newborn compared to adult bones.

We were able to demonstrate that the Yakutian population formed before the 15^th ^century, from a small group of settlers from the Cis-Baïkal region and a small number of women from different South Siberian origins. The genetic characteristics of the Yakuts were well established in the Central Yakutian population during the 15^th ^century, even if there was a small loss in genetic variation during the last two centuries associated with stochastic processes or other phenomena.

## Methods

### Ancient material

The 58 ancient individuals originating from Central Yakutia were excavated over three years in three different districts (*ulus*) (Figure 1). In these areas the winter temperature is less than -40°C for three months and in summer (July and August) the permafrost is located between 30-165 cm under the surface according to our observations, depending on the orientation and the presence/absence of trees which block the sunlight in summer. The graves and the associated artifacts were intact or well preserved (especially wood) and were dated by dendrochronology [57]. The preservation state of the bodies varied from well preserved skeleton remains to naturally mummified bodies, depending on the depth of the graves and/or the presence of overhanging trees. The excavations took place in July and August when the temperature of the graves varied between 1-2°C, and several graves were still frozen upon opening (Figure 2). Sampling took place a few minutes after the graves were opened by a specialist, and some of them were doubled at the autopsy or at the excavation. Bone samples (*e.g*. long bone fragments and/or patella for adults, full long bones for children) were collected from all the subjects (except for YAKa83 in order to preserve the body). Teeth free from cracks or carious lesions and hairs were collected depending on the availability of these two substrates. The dating of the graves was inferred by archaeologists taking into consideration the dendrochronological analyses made on the wood of the graves, radiocarbon ^14^C dating in cases of bad preservation of the wood, and archaeological features such as the artifacts associated with the body; the type of the coffin and the construction of the grave. The samples were distributed across five periods archaeologically dated according to changes in the funerary ritual and the grave artifacts found: the first period spans from the 15^th ^century (the most ancient Yakut graves discovered in Yakutia) to 1689 when the trading post of Nertchinsk between Yakutia and China was created, influencing the traditional Yakutian culture to include many Chinese and/or Mongolian traditions; the second period spans from 1689 to 1728, representing the golden age of Yakutia with Mongolian influences; the third period spans from 1728 to the late 18^th ^century, a cultural decline since a new trading post in Kiakhta partially excluded Yakutia from the travel roads; and the latest period begins from the 19^th ^century (Christianization). The location of the graves, the dating and the type of substrates collected are presented in Additional file 5. Finally, we also analyzed seven samples; six from protohistoric times and one from the 15^th ^century, coming from ancient excavations in Yakutia and deposited in museums and other institutions 3-15 years ago.

### Present day samples

Buccal swabs were taken from 166 volunteers from different regions of Central Yakutia. Informed consent was obtained. The sampling was carefully planned in order to avoid any familial relationship between the donors, the birthplaces of the donors' parents and grandparents were recorded. Autosomal and Y-chromosomal STRs were analyzed employing the same methodologies as for the ancient samples. MtDNA sequencing was conducted at the Institute of Legal Medicine at the Innsbruck Medical University, Austria. All experiments were performed according to EMPOP standard procedures in sequencing and validation in order to ensure a high sequence quality and reliable base calling (full control region [CR] analysis, double amplification of the CR in two separate reactions using different DNA polymerases, sequencing of both strands of the template molecule, minimum double sequencing coverage, automated data transfer, double inspection of data by two independent scientists and final validation by a third) [58]. According to their SNP pattern, samples were assigned to the most terminal haplogroups possible [89]. Data are presented in Additional file 6. On publication, mtDNA sequences will be included in the EMPOP mtDNA database http://www.empop.org.

### DNA extraction and amplification

The outer surface of the bones was abraded to a depth of 2-4 mm using a sanding machine (Dremel, Breda, Netherlands). The decontamination of the teeth was performed as follows: the sample was soaked in deionized and decontaminated water, followed by a brief cleaning with a DNase solution (DNA away MSDS, Molecular Bioproducts, San Diego, USA). The sample was then rinsed in deionized water, soaked in absolute ethanol and finally irradiated under UV light for ten minutes. Bone powder was generated using a column drill and a surgical trepan for long bones. Small pieces of bones and teeth were ground in liquid nitrogen using a Spex 6800 freezer-mill (Metuchen, USA). DNA was extracted from two grams of bone or tooth powder according to the method described by Keyser-Tracqui and Ludes [59]. This protocol includes the overnight incubation at 50°C in a demineralization/lysis buffer, the phenol-chloroform extraction, the purification of the DNA on silica-filter columns (Cleanmix kit, Talent, Trieste, Italy) and finally the concentration of the samples with Microcon^® ^YM30 (Millipore, Billerica, USA). DNA was extracted from hair shafts according to the protocol described by Amory et al. [60].

### Real time PCR quantification

Nuclear DNA quantification was performed on an ABI Prism 7000 SDS (Applied Biosystems, Courtaboeuf, France) using the Quantifiler^® ^Human DNA Quantification Kit (Applied Biosystems, Foster City, USA) according to the manufacturer's protocol. In addition to the quantification of the nuclear DNA, the presence of PCR inhibitors was determined by the co-amplification of an Internal PCR Control included in each reaction.

### Y-chromosomal STRs and SNPs amplification and analysis

Two commercial kits were used for Y-chromosomal STRs amplification. The PowerPlex^®^-Y System (Promega, Madison, USA) which includes 11 STR loci and the AmpF *l*STR^® ^Y-Filer™ kit with 17 loci (Applied Biosystems, Foster City, USA). The PCR amplifications were performed on a T3 Biometra thermocycler (Whatman Biometra, Goettingen, Germany) using the conditions recommended by the manufacturer. Capillary electrophoresis was run on an ABI Prism 3100 system (Applied Biosystems, Foster City, USA) and data analysis performed with the Genemapper software (Applied Biosystems, Foster City, USA).

Since the assignment to haplogroups is not possible on the basis of the STR data only, we analyzed 13 Y-chromosomal SNPs characterizing Asian and Amerindian populations (See result in Additional File 7). These SNPs were amplified and analyzed according to the protocol developed by Bouakaze et al. [61].

The STR haplotypes obtained were individually compared to the YHRD database and to Y-STR data from literature. Molecular diversity indices and Fst genetic distances were calculated for Y-chromosomal STRs data using the ARLEQUIN v2.001 software [62]. Only the nine markers of the "minimum haplotype" (DYS19, DYS389I, DYS389II, DYS390, DYS391, DYS392, DYS393, DYS385a/b) were used as most of the data available for comparison do not include all the markers amplified in our study. A Multi Dimensional Scale (MDS) analysis based on pairwise Fst distances was computed with the software SPSS v13.0 (SPSS Inc., Chicago, USA) (references included in Figure 3). Finally, we constructed a Median Joining (MJ) network based on STR haplotypes belonging to the N1c haplogroup (previously known as N3, [63]) from various Siberian populations (references included in Figure 4). The MJ network computations were realized with Network 4.2.0.1 http://www.fluxus-engineering.com/. The different loci were weighted considering the mutation rates published by Kayser et al. [11].

### MtDNA amplification and analysis

A 421 base pair sequence of the HVS-1 region (from the position 15989 to 16410 of the Cambridge Reference Sequence [64]) was amplified in two overlapping fragments as described by Keyser-Tracqui et al. [47]. The cycle sequencing reaction was performed with the BigDye Terminator v1.1 Cycle Sequencing Kit (Applied Biosystems, Foster City, USA). The products were detected on an ABI Prism 3100 automatic sequencer (Applied Biosystems, Foster City, USA) and analyzed with the Sequence Navigator Software package (Applied Biosystems, Foster City, USA). The haplotypes were assigned to the different haplogroups using the "near matching" method [26] based on the patterns of shared haplogroup-specific or haplogroup-associated polymorphisms of the HVS-1 region. Through this strategy the potential haplogroup status can then be inferred through a motif search and (near-) matching with the sequences used for comparative analysis selected from our database (in the local database of the Institute of Legal Medicine of Strasbourg including 24155 Asian HV1 sequences) and for which (i) haplogroup status has been confirmed with coding-region information in most cases and/or (ii) the full HVS-I haplogroup motif is represented. This allows us to link combinations of HVS-I mutations with certain mutations in the coding region or with sequences harbouring a full HVS-I haplogroup motif and anticipate the haplogroup status of our samples. The potential and utility of the near-matching method has previously been described (e.g. [26]).

The ARLEQUIN software v2.001 [62] was used to calculate genetic diversity and pairwise Fst distances between our ancient sample and 14 populations from Siberia and Central Asia (references included in Figure 5). Pairwise Fst genetic distances were plotted in a MDS graph using SPSS 13.0 (SPSS Inc., Chicago, USA). Finally, migration rates between our ancient Yakut sample and Buryat [4], Evenk [6,65], Mongol [48] and Tuvan [3,6] populations were inferred with MIGRATE [66,67] using coalescence theory. For each population, sequences were selected from different data sets to obtain a sample comprising of approximately 100 individuals, in order to limit the bias linked to heterogeneous sample sizes. The parameters were set as recommended by Brandstätter et al. [68] (i.e. ten short and two long chains were run, each with a short sampling increment of 20, the number of discarded trees per chain ["burn-in"] was set to 10,000, the search was run with four chains at different temperatures (1.0, 1.2, 1.5, and 3.0) with an "adaptive heating" scheme).

### Measures against contaminations and validation of the data

Bearing in mind the critical issues of pre-laboratory contaminations encountered in ancient DNA studies [69], all the excavations were performed with extensive precautions using face masks and latex gloves. Bones, teeth and hairs were collected by a trained scientist. Samples were stored for a short period in sealed plastic bags under the appropriate conditions before being transferred to the laboratory, where they were stored at -20°C until analysis. Moreover, the samples underwent no prior treatments before laboratory analyses, to avoid possible contamination and to prevent deleterious effects on the DNA [31]. A comparison was done between morphological and molecular sex typing methods.

In addition to the Y chromosome and mtDNA, autosomal STRs were analyzed due to their high discrimination power, and results were systematically compared with the profiles of the archaeologists involved in the excavations and the two scientists leading the DNA extractions. DNA profiles of each ancient specimen are given in Additional file 4. Indeed, this comparison of the results obtained for the ancient samples and the genotypes of the researchers is considered as a key for the validation of data [47,69,70]. The precautions concerning the facilities, the laboratory equipment and the reagents were thoroughly respected (laboratory dedicated to ancient DNA only, strict separation of pre- and post-PCR areas, UV irradiation of the rooms and the laboratory equipment between each experiment, multiple DNA extractions and PCR amplifications, systematic use of negative controls, etc.). The comparison of the results obtained from different substrates for the same individual was used in this study as an additional validation step. The DNA extractions, separated in time, from different types of tissue, reinforce the possibility to identify potential contaminations in one of the DNA extracts since it is unlikely that a contaminant would spread to all of them. Moreover, the comparison of the results obtained from these different extracts is extremely useful to highlight allelic dropouts, spurious alleles or point heteroplasmies.

*A posteriori *data quality control was performed applying the NETWORK software that is freely available from the EMPOP website http://www.empop.org. By filtering speedy or even all known mutations from a dataset, this program is able to pinpoint potential errors in a data set among the remaining mutations (e.g. caused by mistakes in transcription, artificial recombination, phantom mutations). All these mutations in the Yakuts dataset were carefully inspected and verified. The parameters for this analysis were set as recommended by [71]. An independent laboratory repeated mtDNA analyses for three of the samples and found the same results as we did [72].

### First presence of the Yakutian ancestors and demographic data

Our surveys and excavations focused specifically on ancient graves. At the same time, we developed a research program to estimate the number of Yakut people in the middle of the 17^th ^century according to the tax collectable in pelt imposed by Russians (Ϊassak). The number which seems the most probable is approximately 40,000 including children (Gogolev, personal communication). We postulated that this number results from a demographic expansion and we tried to reconstruct its origin by a simulation using logistic regression [73] with a growth coefficient/year of between 0.6% to 3%. The first percentage is typical for animal-breeding farmers in new territories, for example it was 1% for the Vikings when they arrived in Iceland in the 9^th ^century [9]; the last seems too high on the long term but not impossible over a short period [74].

## Abbreviations

aDNA: (ancient DNA); mtDNA: (mitochondrial DNA); HVS-1: (Hyper-variable segment 1); PCR: (Polymerase chain reaction); STRs: (Short Tandem Repeats); MDS plot: (Multidimensional scaling plot); SNPs: (Single Nucleotide Polymorphisms).

## Authors' contributions

LB and CE, conceived and designed the research protocol. CE and AS conducted the field study. LB, KC, AS, BC, conceived and designed the experiments. AS, BC, KC, performed the experiments. AS analyzed the data. CE and AS wrote the paper. GM selected and provided a part of present day Yakuts samples; PW, BM, RA contributed to the mtDNA analysis and the editing of the manuscript.

All authors read and approved the final manuscript.

## Supplementary Material

Additional file 1 **Table of the consensus Y-STR haplotypes determined for the ancient Yakut samples**. Haplogroups were defined by Y SNP snapshot typing detailed in Additional file 7. Samples with grey cells correspond to samples typed by the PowerPlex^®^-Y System. Alleles in parentheses correspond to alleles missing in one of the amplifications for a given sample. Click here for file

Additional file 2 **MtDNA HVS-1 polymorphisms and haplogroups (Hg) of the ancient Yakut sample**. List of the mtDNA haplogroups of the ancient Yakut sample. rCRS: revised Cambridge reference sequence. Click here for file

Additional file 3 **mtDNA Haplotypes shared between the ancient Yakut and Siberian populations**. List of haplotypes shared between the present data ancient Yakut and the Siberian populations. Populations are coded as follow. Yak: present day Yakuts, Bur: Buryats [3,4] (Shimada 2001 unpublished), Chuk: Chuckches [85,86], EG_PD: Eygin Gol present day [48], EG_A: Eygin Gol ancient [47], Esk: Eskimos [85,86], Evk: Evenks [50,65,87], YSE: Yakut Speaking Evenks [6], Kaz: Kazakhs [23,26], Kir: Kirghiz [23], Kor: Koryaks [83], Man: Mansis [22], Mg: Mongols [26,48,65,88], Oro: Oroquens [65], Tuv: Tuvans [3], Uyg: Uyghurs [23], Uzb: Uzbeks [84], Yuk: Yukhagirs [6]. Click here for file

Additional file 4 **Consensus autosomal STR profiles determined for the ancient Yakut samples**. Consensus autosomal STR profiles determined for the ancient Yakut samples. Samples with grey cells correspond to sample typed by the the AmpF STR Profiler Plus kit. Click here for file

Additional file 5 **Localization, dating and substrate(s) collected for the different individuals**. The different ancient samples collected. Dendro: dendrochronological dating, Archaeo: archaeological dating. I: incisive tooth, C: canine tooth, pM: pre-molar tooth, M: molar tooth. Data obtained from samples in italic font were not taken into account for the phylogenetic analyses. Click here for file

Additional file 6 **MtDNA control region polymorphisms of the 166 present day Yakuts**. MtDNA control region polymorphisms. Haplogroups assigned according to van Oven and Kayser [89] mtDNA tree build 5. Click here for file

Additional file 7 **Y SNP polymorphisms of ancient Yakut samples**. 13 Y-chromosomal SNPs typing of the ancient Yakut samples. These SNPs were amplified and analyzed according to the protocol developed by Bouakaze et al. [61]. Click here for file
